# Antimicrobial mechanical and molecular docking analysis of dental composite resin incorporating green synthesized titanium dioxide nanoparticles from *Vitis vinifera* extract

**DOI:** 10.1038/s41598-025-20989-5

**Published:** 2025-10-08

**Authors:** Dina Abozaid, Mohamed Abd El-Aal, Zienab E. Eldin, Mohamed Abdelmonem, Mohamed Ahmed Ibrahim, Abdulrahman M. Saleh, Amr Azab

**Affiliations:** 1 Dental Biomaterials Department, Faculty of Dentistry, Tanta University, Tanta, 31511 Egypt; 2 Chemistry Department, Faculty of Science, Assiut University, Assiut, 71516 Egypt; 3 Materials Science and Nanotechnology Department, Faculty of Postgraduate Studies for Advanced Sciences, Beni-Suef University, Beni-Suef, 62511 Egypt; 4 Department of Pharmaceutics and Industrial Pharmacy, Faculty of Pharmacy, October University for Modern Sciences and Arts (MSA), Giza, 12451 Egypt; 5 Department of Pharmaceutical Chemistry, Faculty of Pharmacy, Cairo University, KasrEl- Aini Street, Cairo, 11562 Egypt; 6 Infection Control and Epidemiology Surveillance Unit, Aweash El-Hagar Family Medicine Center, Ministry of Health and Population (MOHP), Mansoura, 35711 Egypt; 7 Prosthodontics Department, Faculty of Dentistry, Tanta University, Tanta, Egypt

**Keywords:** Green-synthesized TiO₂ nanoparticles, *Vitis vinifera*, Dental resin composite, Antibacterial activity, Polymerization shrinkage, Molecular docking interaction, Computational biology and bioinformatics, Drug discovery, Microbiology, Molecular biology, Plant sciences, Health occupations, Nanoscience and technology, Health care, Dental materials, Dental public health, Infection control in dentistry, Preventive dentistry, Restorative dentistry

## Abstract

Dental composites are widely used restorative materials but secondary caries to the restored tooth, polymerization shrinkage, and mechanical failure, limiting their longevity. Incorporating nanoparticles synthesized via eco-friendly methods offers a promising strategy to enhance composite performance. This study introduces a novel, eco-friendly strategy to enhance dental composite resins by incorporating TiO₂-NPs synthesized using *Vitis vinifera* extract. This study uniquely employs *Vitis vinifera*, rich in flavonoids and phenolics, as a reducing and capping agent, and for the first time integrates molecular docking analysis to explore the mechanistic antimicrobial interaction between TiO₂-NPs and *Streptococcus mutans* glucosyltransferase. The research evaluates the impact of these green-synthesized nanoparticles on antimicrobial properties, mechanical performance, polymerization shrinkage, and their molecular interactions with *Streptococcus mutans* glucosyltransferase via molecular docking analysis. The green-synthesized TiO₂-NPs were thoroughly characterized and integrated into dental composites at 10% and 20% by weight (Groups II and III, respectively), while Group I (control) contained only fumed silica fillers. The antimicrobial activity was assessed against *S. mutans*,* S. sanguinis*,* and L. acidophilus*, and mechanical properties (flexural strength and microhardness) were evaluated according to ISO standards. Polymerization shrinkage was measured using a strain gauge. Additionally, molecular docking analysis was performed to investigate the interaction of TiO₂-NPs with *S. mutans* glucosyltransferase. The modified composites exhibited significantly improved antimicrobial activity against key oral pathogens and superior mechanical properties compared to the control. Molecular docking studies provided insights into the potential antimicrobial mechanisms of TiO₂-NPs. These findings suggest that green-synthesized TiO₂-NPs offer a promising, eco-friendly approach to improve the performance and longevity of dental restorative materials.

**Supplementary Information:**

The online version contains supplementary material available at 10.1038/s41598-025-20989-5.

## Introduction

Dental composite resins have become a fundamental material in restorative dentistry due to their aesthetic appeal and adaptability^1^. However, despite significant advancements, conventional dental composites still face critical challenges that limit their long-term clinical success. These include polymerization shrinkage leading to marginal gaps, susceptibility of the restored tooth to secondary caries caused by bacterial colonization, and inadequate mechanical durability under oral stresses^2,3^. Such limitations often result in restoration failure, necessitating replacement procedures that increase patient discomfort and healthcare costs.

To overcome these drawbacks, researchers have explored the incorporation of nanoparticles into dental resins to enhance their antimicrobial properties and mechanical performance^4^. Titanium dioxide nanoparticles (TiO₂-NPs) have attracted considerable attention due to their biocompatibility, photocatalytic, antimicrobial activity, chemical stability, and mechanical reinforcement potential^4^. TiO_2_ is widely used as a white pigment in paints, plastics, and cosmetics, as a UV blocker in sunscreens, and as a photocatalyst for self-cleaning surfaces and pollution control. It’s also used in solar cells, ceramics, food coloring, textiles, and some electronic devices due to its optical and chemical properties^5–8^. Nonetheless, traditional chemical and physical synthesis methods of TiO₂-NPs often involve toxic reagents, high energy consumption, and environmental concerns, which hinder their widespread biomedical application.

Green synthesis of nanoparticles using plant extracts has emerged as a sustainable and eco-friendly alternative, offering advantages such as cost-effectiveness, reduced toxicity, and the presence of natural reducing and stabilizing agents. Among various plant sources, *Vitis vinifera* (grape) belongs to the *Vitaceae* family and is widely consumed globally. Grape seeds are a rich source of polyphenols, flavonoids, and other bioactive compounds, which have demonstrated potent antioxidants and antimicrobial properties^9^. These components act as free radical scavengers and show higher antioxidant activity compared to traditional antioxidants like vitamins C, E, and β-carotene^9,10^.

Furthermore, *Vitis vinifera* has shown promise in the green synthesis of nanoparticles, acting as both a reducing and capping agent during the process^10–12^. Previous studies have demonstrated the successful green synthesis of TiO₂-NPs using *Vitis vinifera* extract, yielding nanoparticles with controlled size, enhanced stability, and promising photocatalytic properties^13–16^. However, the application of such green-synthesized TiO₂-NPs in dental composite resins remains underexplored.

Most earlier studies on TiO₂-NPs incorporated dental composites have focused on commercially available nanoparticles or chemically synthesized by the authors. These studies reported significant antimicrobial activities especially against *S. mutans* and improvements in mechanical properties such as flexural strength and microhardness at various concentrations of TiO₂-NPs^17–19^. However, only a limited number have explored green-synthesized alternatives, and even fewer have directly compared plant-mediated nanoparticles in dental applications.

A recent study by Ezzat et al.^20^ reported the green synthesis of TiO₂ nanoparticles mediated by grapefruit seed extract (GSE) and their incorporation into experimental dental composites. GSE-TiO₂-NPs enhanced antibacterial activity against *S. mutans*, improved flexural strength and modulus, and reduced polymerization shrinkage compared to unmodified composites.

Building upon this foundation, the present study employs *Vitis vinifera* extract for the green synthesis of TiO₂-NPs and incorporates them into dental resin composites. While both grape and grapefruit seed extracts serve as effective green reducing agents, *Vitis vinifera* extract is distinguished by its unique phytochemical profile, which may influence nanoparticle characteristics such as size distribution, surface charge, and bioactivity. By comparing the present findings with those of GSE-mediated TiO₂-NPs composites, this study aims to highlight the advantages of *Vitis vinifera*-mediated synthesis in producing stable TiO₂-NPs that enhance the antimicrobial and mechanical performance of dental composites.

This comparative approach underscores the potential of different plant extracts in tailoring nanoparticle properties for specific dental biomaterial applications and advancing the development of next-generation restorative materials. Thus, green-synthesized TiO₂-NPs, particularly those derived from *Vitis vinifera* extract, have the potential to overcome the most pressing clinical challenges of current dental composites by improving antimicrobial efficacy, mechanical strength, and reducing polymerization shrinkage in a sustainable manner.

The novelty of this study lies in the use of *Vitis vinifera* extract, characterized by its unique phytochemical composition, as a green synthesis medium for TiO₂-NPs, the incorporation of these biogenic TiO₂-NPs into experimental dental composites with validated improvements in antimicrobial, mechanical, and polymerization properties, and the first-time application of molecular docking analysis to elucidate the mechanistic interaction between TiO₂-NPs and a key bacterial virulence enzyme (glucosyltransferase from *S. mutans*), linking physicochemical properties with biological function. This integrated approach offers a deeper understanding of bioactivity mechanisms and sets this work apart from prior studies relying solely on empirical observations.

## Materials and methods

### Materials

The materials that used in this study are listed in Table 1.

**Table 1:** List of materials and their suppliers used in this study.

Materials	Suppliers
Bisphenol A glycidyl methacrylate (Bis-GMA) with a density of 1.161 g/mL at 25 °C	Sigma Aldrich, US
Triethylene glycol dimethacrylate (TEGDMA, 99%) contains 200 ppm of monomethyl ether of hydroquinone with a density of 1.092 g/mL at 25 °C.	
Camphorquinone (CQ) (97%)	
2- (N, N-dimethylamino) ethyl methacrylate (DMAEMA, 98%) containing 700–1000 ppm of monomethyl ether of hydroquinone	
Fumed Silica nanoparticles	
γ-methacryloxypropyltrimethoxysilane (MPTMS, 98%)	
Acetone	
Trypticase Soy Broth media	
Titanium isopropoxide (C₁₂H₂₈O₄Ti, 97%)	
*Streptococcus mutans* (*S. mutans*, ATCC 25175), *Streptococcus sanguinis* (*S. sanguinis*, ATCC 10556), and *Lactobacillus acidophilus* (*L. acidophilus*, ATCC 4356)	VACSERA (Giza, Egypt)
Grape seeds	Grown and harvested during the spring of 2023 in Nubaria
Strain Gauge	)Kyowa, Ltd., Lot #Y4003S, Japan (1 mm in length with an electric resistance of 119.6 ± 0.4 Ω and a gauge factor of 2.13% ± 1.0%

### Preparation of the *Vitis vinifera* extract

Grape seeds were air-dried for two weeks to preserve their active components, followed by vacuum drying at 40 °C using calcium fluoride as a desiccant. The dried seeds were ground into a fine powder using an electric blender. Twenty grams of the powdered seeds were boiled in 200 mL of distilled water for 2 h. The extract was then cooled, filtered through multiple layers of filter paper to remove solid residues, and stored in a refrigerator until further use^21^.

### Green synthesis of titanium dioxide nanoparticles (TiO_2_-NPs)

Titanium dioxide nanoparticles were synthesized by adding 100 mL of titanium isopropoxide solution (97%) dropwise to the grape seed extract while stirring vigorously at 80 °C. The mixture was aged for two hours to ensure uniform particle size distribution. The resulting yellowish-white precipitate was separated via centrifugation at 5,000 rpm for 10 min, washed with distilled water, and centrifuged again. The cleaned precipitate was dried overnight at 80 °C and calcined at 500 °C to yield TiO_2_-NPs^22^. The yield of TiO₂ NPs, determined from the dry weight following calcination, was found to be 95%.

### Preparation for experimental composite resin

The resin matrix comprised equal parts (50 wt%) of Bis-GMA and TEGDMA. Photoinitiators CQ and DMAEMA were added at 0.2 wt% and 0.8 wt%, respectively. The experimental composite was prepared by incorporating 28 wt% of the resin matrix with 72 wt% of fillers (Fumed silica and TiO_2_-NPs). fillers were salinized with 5 wt% MPTMS before being blended with the resin using a Speed Mixer (DAC 150 FVZK, Hauschild Engineering, Germany) at 3,500 rpm for one minute^23,24^. This mechanical mixing was followed by ultrasonication using a probe sonicator (Branson Ultrasonics, 40 kHz) for 10 min to break up any nanoparticle agglomerates and promote uniform distribution. The dispersion quality was visually inspected and further confirmed by scanning electron microscopy (SEM) imaging of the composite cross-sections, which showed well-distributed nanoparticles without significant aggregation.

#### Sample preparation and polymerization

Composite resin samples were prepared by placing the nanoparticle-modified resin into Teflon molds of appropriate dimensions depending on the test. The resin was polymerized using an LED curing light (Bluephase, Ivoclar Vivadent) with an intensity of 1200 mW/cm² for 40 s on each side of the sample. Samples were stored in distilled water at 37 °C for 24 h before testing. Specimen groups were categorized as follows:

1. Group I (Control): Unmodified composite containing only fumed silica fillers.
2. Group II: Experimental composite containing 10% TiO_2_-NPs.
3. Group III: Experimental composite containing 20% TiO_2_-NPs.

The TiO₂-NP concentrations of 10 wt% and 20 wt% were selected based on prior studies that demonstrated significant improvements in antibacterial and mechanical properties of dental composites at these levels without adversely affecting the curing process or material integrity^4,20^.

In all composite formulations, the total filler content was consistently maintained at 72 wt%. For the control group, the filler system consisted solely of fumed silica. In the experimental groups, part of the fumed silica was substituted with green-synthesized TiO₂ nanoparticles. Specifically, 10 wt% and 20 wt% of the fumed silica was replaced with TiO₂-NPs while keeping the overall filler loading constant at 72 wt%. This approach ensured that differences in composite performance could be attributed to the presence of TiO₂-NPs rather than variations in total filler content.

### Characterization techniques

The *Vitis vinifera* extract was chemically analyzed using a GC-MS system equipped with a polar HP-5ms column. Extracts were diluted in acetone prior to injection. The analysis was conducted with helium as the carrier gas, an injector temperature of 200 °C, and a detector temperature of 250 °C^25^.The crystal structure and phase identification of the synthesized TiO_2_-NPs was observed using X-ray diffraction spectroscopy (XRD) utilizing SHIMADZU XRD-6000 (Shimadzu Corporation, Kyoto, Japan) technique under the conditions power diffraction system.

The target was Cu-Ka x-ray tube with a wavelength (λ = 1.5406 Å). The X-ray scan was performed between 2*θ* equal to 4° and 80°. The voltage was 40 KV and the current was 30 mA, the scan mode was continuous, the speed was 5.0000 (deg. /min) and was handled by x-pert software^26^. The average crystallite size was determined using the Scherrer equations^27–29^. The modified composite resin was morphologically characterized using Scanning electron microscope Morphology was observed using a JEOL JSM-6510LV scanning electron microscope (JEOL Ltd., Tokyo, Japan)^30^.

FTIR spectra were recorded using a PerkinElmer Spectrum Two FTIR spectrometer (PerkinElmer Inc., Waltham, MA, USA). Spectra were recorded with a resolution of 1 cm^−1^, comprising ten scans across the wavenumber range of 4000 to 400 cm^−1^.

The average hydrodynamic size, particle size distribution and polydispersity were assessed using the Dynamic Light Scattering (DLS) technique with a ZS90 Zetasizer instrument from Malvern, UK. The Zeta potential, which indicates the colloidal stability, was measured through electrophoretic laser Doppler velocimetry^31^. Morphological characteristics of the nanoparticles were observed using a JEOL 2100 F TEM. Samples were prepared on copper grids after dispersion in distilled water and sonication^32^.

### Evaluation of experimental composite resin

#### Antimicrobial activity measurement

The antimicrobial efficacy of composite resins was rigorously evaluated using a standardized agar diffusion method against three key oral bacterial strains: *Streptococcus mutans (S. mutans*, ATCC 25175), *Streptococcus sanguinis (S. sanguinis*, ATCC 10556), and *Lactobacillus acidophilus (L. acidophilus*, ATCC 4356). All bacterial strains were procured from VACSERA (Giza, Egypt)^33^.

*S. mutans* and *S. sanguinis* were cultivated on Mitis Salivarius agar (Difco, USA) supplemented with 0.2 units/mL bacitracin to ensure selective growth. Cultures were incubated aerobically at 37 °C in a humidified atmosphere containing 5% CO₂ for 24 h. *L. acidophilus* was grown on de Man, Rogosa, and Sharpe (MRS) agar (Oxoid, UK) under strict anaerobic conditions (AnaeroPack System, Mitsubishi Gas Chemical, Japan) at 37 °C for 24 h. Following incubation, bacterial cells were harvested and suspended in sterile physiological saline solution (0.9% NaCl). The optical density of each bacterial suspension was adjusted to match a 0.5 McFarland standard, corresponding to approximately 10⁸ colony-forming units per milliliter (CFU/ml) for all strains. This standardization was verified spectrophotometrically at 600 nm and confirmed by plating serial dilutions on appropriate agar media.

Mueller-Hinton agar (Oxoid, UK) plates, with a uniform thickness of 4 mm (approximately 25 mL per 90 mm diameter Petri dish), were prepared according to manufacturer instructions and sterilized by autoclaving. Each agar plate was inoculated with 100 µL of the standardized bacterial suspension (10⁸ CFU/ml). The inoculum was evenly spread across the entire surface of the agar using a sterile L-shaped glass spreader, ensuring a confluent lawn of bacterial growth. The inoculated plates were allowed to dry for 15 min at room temperature prior to disc placement^34^.

Disc-shaped specimens of the composite resins, each measuring 4 mm in diameter and 2 mm in thickness, were prepared under aseptic conditions. All specimens were sterilized by exposure to ultraviolet (UV) light for 30 min on each side prior to placement on the inoculated agar plates. A total of five replicate specimens were used for each composite resin type against each bacterial strain. These specimens were aseptically placed on the surface of the inoculated agar, ensuring firm contact without embedding.

For robust comparative analysis, two control groups were included on each agar plate. Sterile paper discs (6 mm diameter, Whatman No. 1 filter paper) were impregnated with 20 µL of 0.12% chlorhexidine (CHX) solution (Sigma-Aldrich, USA) and allowed to air dry for 10 min before placement. CHX is a well-established antimicrobial agent, serving as a benchmark for efficacy.

Sterile paper discs (6 mm diameter, Whatman No. 1 filter paper) were impregnated with 20 µL of 10% dimethyl sulfoxide (DMSO) solution (Fisher Scientific, USA), which was used as the solvent for certain components in the composite resins. DMSO served as a vehicle control to ensure that any observed antimicrobial activity was attributable to the composite resin itself and not to the solvent.

Both control discs were placed on the same agar plates as the composite resin specimens, maintaining adequate spacing to prevent overlapping zones of inhibition.

All inoculated plates, including those with specimens and controls, were incubated at 37 °C under strict anaerobic conditions (AnaeroPack System, Mitsubishi Gas Chemical, Japan) for 24 h. This consistent anaerobic environment was maintained for all bacterial strains to ensure optimal growth conditions for the tested oral pathogens and to standardize the experimental setup. After 24 h of incubation, the plates were visually inspected for zones of inhibition around each specimen and control disc. The diameter of the clear zone of inhibition (in millimeters), including the diameter of the disc/specimen, was measured using a digital caliper (Mitutoyo, Japan). Measurements were taken at three different points for each zone, and the average diameter was recorded. The presence and size of the inhibition zone indicated the antimicrobial activity of the tested material. All measurements were performed in triplicate for each specimen, and the mean and standard deviation were calculated.

#### Molecular docking measurement

To investigate the potential molecular mechanism underlying the antimicrobial activity of green-synthesized TiO₂ nanoparticles (TiO₂-NPs), molecular docking study was performed targeting the catalytic domain of *S. mutans* glucosyltransferase (GtfC), a key enzyme in biofilm formation and caries pathogenesis. The three-dimensional structure of GtfC was retrieved from the Protein Data Bank (https://www.rcsb.org/) with PDB ID : 8fjc^35^. The protein structure was prepared by removing water molecules and adding hydrogen atoms. Given the inorganic and particulate nature of TiO₂-NPs, a representative cluster model of anatase-phase TiO₂ was constructed based on the dominant crystallographic facets observed in TEM analysis, following established modeling approaches for metal oxide nanoparticles. The TiO₂-NPs was energy-minimized using the Universal Force Field (UFF) in Avogadro software to obtain a stable geometry suitable for docking^36–38^.

Docking simulation was performed using AutoDock Tools 1.5.6., with the grid box centered on the active site of GtfC as defined by co-crystallized ligands and key catalytic residues. The binding affinity (ΔG, kcal/mol) and interaction profiles were analyzed, focusing on the interactions between the TiO₂-NPs cluster and the enzyme’s active site residues. During docking process, twenty different poses were created with the protein target site, Then the best-fitted pose with the active site was recorded and 3D figure was generated by the Discovery Studio 2024 visualizer.

#### Flexural strength (FS) measurement

Bar-shaped specimens (2 × 2 × 25 mm) were tested using a three-point bending test on a universal testing machine (INSTRON, 3345 series, Norwood, MA, USA). Specimens were loaded at a crosshead speed of 0.75 mm/min until fracture occurred^39^. After the specimens were set, they were taken out, and the extra material was ground off using 800-grit silicon carbide paper. All specimens were then placed in distilled water and incubated at 37 °C for 24 h. The flexural strength was measured using a three-point bending method after the test assembly was attached to a universal testing machine (Instron 3345, Instron Corp., Norwood, MA, USA) with a crosshead speed of 1 mm/min and a span of 20 mm^40^. To calculate flexural strength, the following formula was used:

1 $${\mathbf{FS}} = {\mathbf{3F}}{\text{ }}({\mathbf{l}})/{\text{ }}{\mathbf{2wh}}^{2}$$

where **F** is the load at fracture, **l** is the distance between the supports (20.0 mm), **w** is the specimen width, and **h** is the specimen height^41^.

#### Vickers microhardness measurement

For Vickers microhardness testing, disc-shaped specimens (6 mm in diameter and 2 mm thick) were prepared. Once the specimens had set, they were carefully removed from their molds and polished with 800-grit silicon carbide paper to standardize the surface and eliminate any excess material. Following polishing, the specimens were stored in distilled water at 37 °C for 24 h. to simulate pre-testing storage conditions^42^. After the storage period, the specimens were air-dried and tested with a Digital Display (Model HVS-50, Laizhou Huayin Testing Instrument Co., Ltd., Laizhou, China) equipped with a Vickers diamond indenter and a 20× objective lens. A consistent load of 100 g was applied for 15 s. To ensure accuracy and reproducibility, three indentations were made on each specimen, evenly spaced by at least 0.5 mm. The Vickers hardness values were calculated using the standard formula^43^:

2 $${\mathbf{VHN}} = {\mathbf{1}}.{\mathbf{854}}{\text{ }}{\mathbf{P}}/{\mathbf{d}}^{{\mathbf{2}}}$$

where VHN is Vickers hardness in Kgf/mm^2^, P is the load in Kg and d is the length of the diagonals in mm.

#### Polymerization shrinkage measurement

Post-gel polymerization shrinkage was measured using a strain gauge method. Composite specimens were cured in Teflon mold with strain gauges attached to a flat glass surface in biaxial configuration. The strain gauges (Kyowa, Ltd., Japan) were calibrated prior to testing using a standard reference material with known shrinkage characteristics to ensure accuracy and sensitivity. All measurements were conducted in a temperature-controlled environment at 23 ± 1 °C and 50 ± 5% relative humidity. The specimens were cured using an LED light source (1200 mW/cm²) for 40 s, consistent with the curing protocol used in mechanical testing^44^. The shrinkage strain was recorded continuously using a digital strain meter (Kyowa PCD-300 A, Japan), and average values were calculated from three replicates per group.

The unmodified composite (Group I) served as a control to assess the baseline polymerization shrinkage associated with the fumed silica-based resin. Modified groups (10% and 20% TiO₂-NPs) were compared accordingly.

### Statistical analysis

Statistical analysis was performed using IBM SPSS Statistics version 29.0 (IBM Corp., Armonk, NY, USA). Data distributions were evaluated for normality with the Shapiro–Wilk test and for homogeneity of variance with Levene’s test. When both assumptions were satisfied, inter-group differences were examined using one-way ANOVA with Tukey post-hoc comparisons; if variances were unequal, Welch’s ANOVA with Games–Howell post-hoc tests was employed. Datasets departing from normality were analysed with the Kruskal–Wallis test, followed by Bonferroni-adjusted pairwise comparisons. All results were considered statistically significant at *p* < 0.05.

**Fig. 1:**
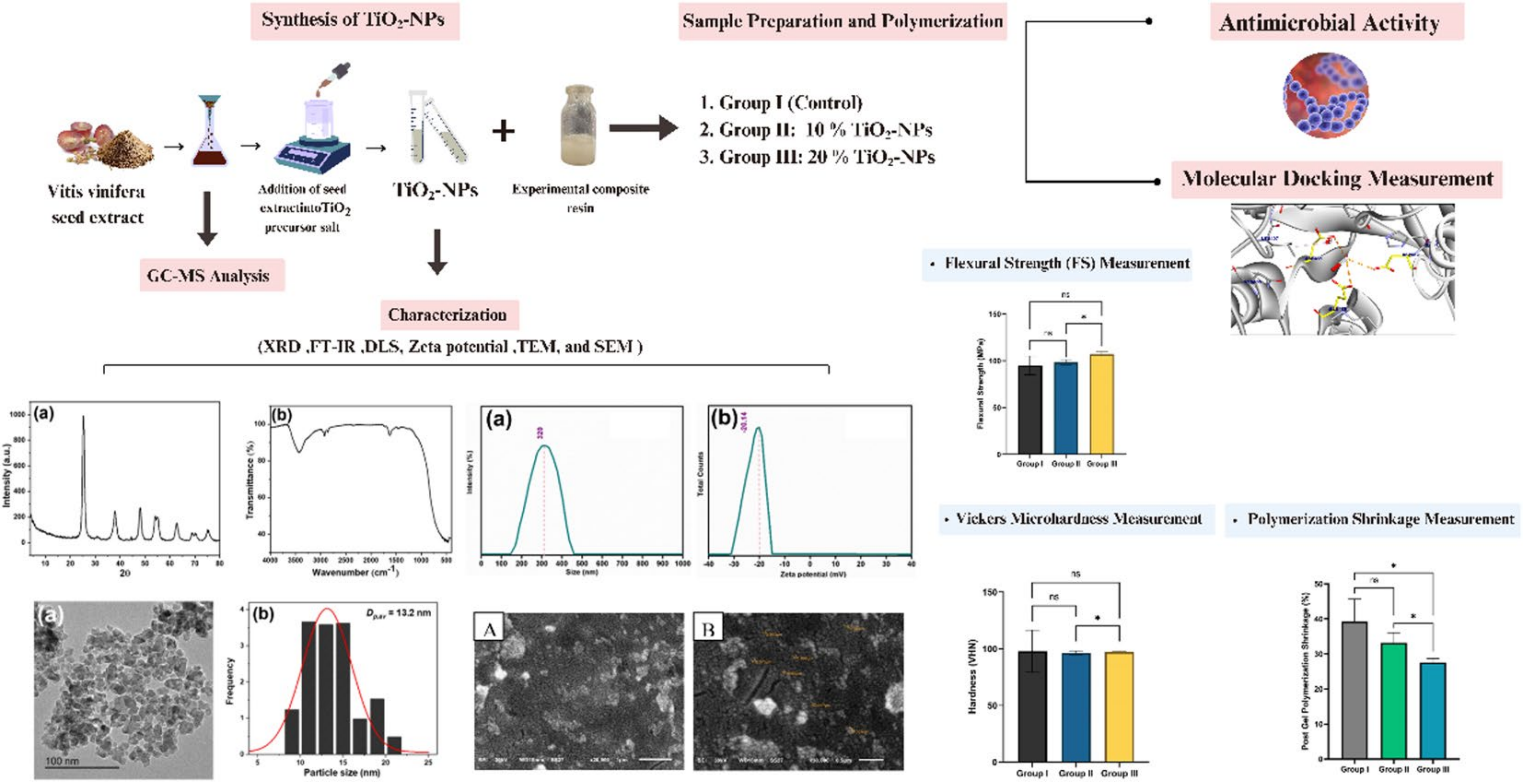
A schematic representation of methodological steps.

A schematic representation of the methodological steps is shown in Fig. 1.

## Results

### Characterization analysis

The *Vitis vinifera* extract contains a diverse range of bioactive compounds spanning multiple chemical classes, such as fatty acids (e.g., oleic acid, linoleic acid, stearic acid), phenolic compounds (e.g., cinnamic acid, eugenol), and terpenes (e.g., myrcene, β-caryophyllene). These constituents are likely responsible for the extract’s notable bioactivity, including antioxidant, anti-inflammatory, and potential antimicrobial properties. Table 2 summarizes the top 30 identified compounds, detailing their retention time (RT), names, and normalized area percentages (Norm %).

**Table 2:** GC-MS analysis of *Vitis vinifera* extract.

No.	RT (min)	Compound name	Norm %
1	9.745	Phenylethanol (Phenylethyl Alcohol)	0.56
2	12.181	1,2-Benzenediol (Catechol)	0.51
3	12.236	Resorcinol	1.03
4	12.381	Metacetamol	1.84
5	12.516	Histidine	0.54
6	12.566	Hydroquinone	0.52
7	12.601	Propoxur	0.91
8	12.781	n-Decyl(phenyl)sulfide	0.88
9	12.976	Hydroquinone	0.87
10	13.076	1,3-Benzenediol, 4,4’-thiobis-	0.66
11	13.146	Resorcinol	0.51
12	13.316	Hydroquinone	0.57
13	13.366	Histidine	0.61
14	13.446	1,2-Benzenediol (Catechol)	0.70
15	13.556	4(1 H)-Pyrimidinone, 6-methyl-	0.59
16	13.707	Disperse Blue 26	0.58
17	15.817	1,2,3-Benzenetriol	15.49
18	16.183	Vanillin	3.83
19	18.799	2-Methyl-9-α-d-ribofuranosylhypoxanthine	0.51
20	19.374	1,3-Benzenediol, 4-propyl-	47.44
21	19.794	4-tert-Butylcatechol	8.52
22	20.164	Benzene-1,2-diol, 4-(2-guanidinothiazol-4-yl)-	0.79
23	20.224	Cyclohexane, 1,1’-hexylidenebis-	0.59
24	20.254	Butanal, (2,4-dinitrophenyl)hydrazone	0.64
25	20.419	Hexadecenoic acid, Z-11-	1.03
26	21.425	Tetradecanoic acid (Myristic Acid)	0.97
27	22.030	2-Hydroxy-5-methylisophthalaldehyde	4.01
28	24.791	n-Hexadecanoic acid (Palmitic Acid)	100.00
29	28.012	Octadecenoic acid	8.89
30	28.678	Hexadecanoic acid derivative	40.34

**Fig. 2:**
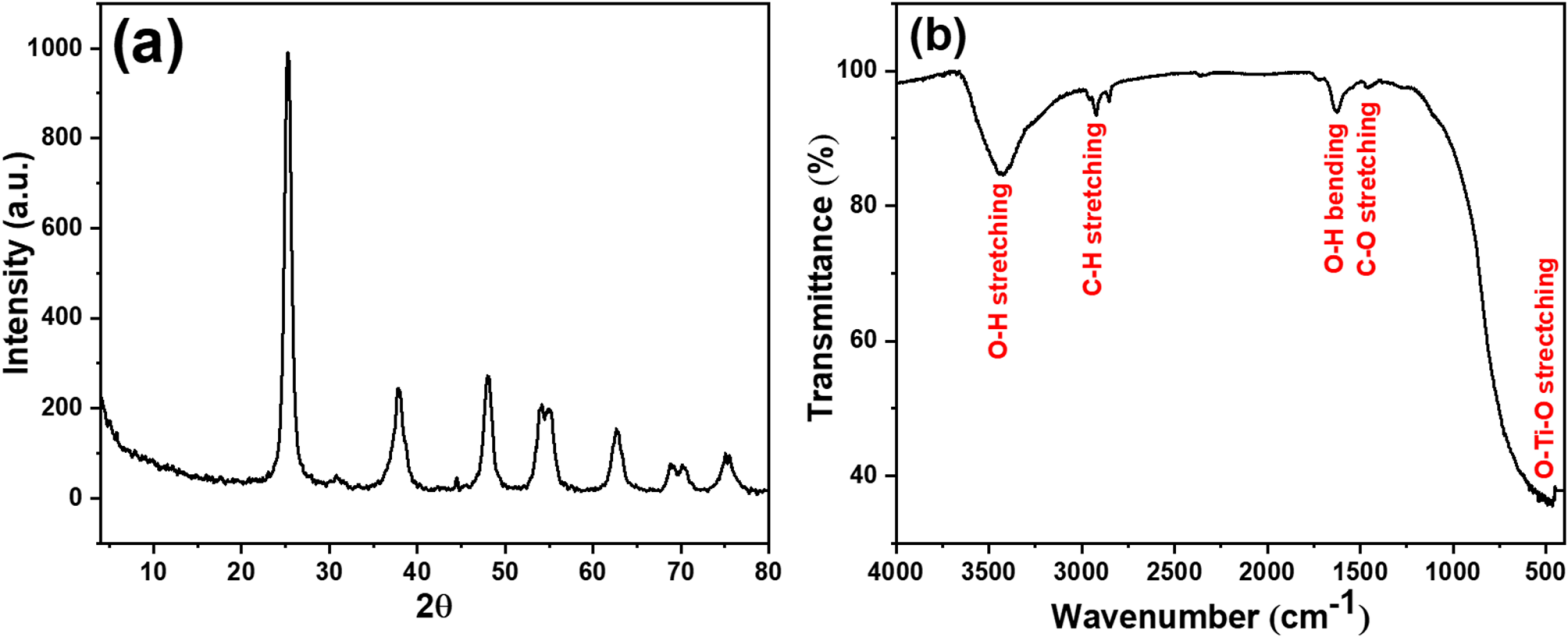
(**a**) XRD pattern and (**b**) FTIR spectrum of TiO_2_-NPs.

The XRD pattern for the TiO_2_-NPs sample is shown in Fig. 2a. It showed diffraction peaks at 2*θ* angles of 25.3°, 37.9°, 48.2°, 54.1°, 55.2°, 62.7°, 69.1°, 70.4°, and 75.4°. These peaks correspond to the (101), (004), (200), (105), (211), (204), (116), (220), and (215) planes of tetragonal anatase TiO_2_ (JCPDS No. 01-084-1286). The average crystallite size was determined using the Scherrer equation^27–29^, yielding a value of 12.7 nm. The crystallinity of the TiO₂ NPs, as estimated from XRD peak intensities and area analysis, was found to be 72%, indicating a predominantly crystalline anatase phase.The obtained XRD results are consistent with those reported by Yitagesu et al.^45^, who synthesized anatase TiO_2_ with a tetragonal crystal structure and an average crystallite size of 11 nm using *Impatiens rothii* Hook.f. leaf extract. TiO_2_ NPs synthesized using *Luffa acutangula* leaf extract were found to possess a rutile phase with a face-centered cubic (FCC) crystal structure^46^. TiO_2_ NPs synthesized via the green method using jasmine flower extract were found to exhibit a rutile phase with a tetragonal crystal structure and an average crystallite size ranging from 31 to 42 nm^47^. TiO_2_ NPs synthesized using mulberry plant extract exhibited an anatase phase with a crystallite size of 24 nm^48^. The XRD pattern of TiO_2_ NPs synthesized via the green method using *Echinops echinatus* plant extract revealed an anatase phase with an average crystallite size of 13 nm^49^.

Figure 2b presents the FTIR spectrum for TiO_2_-NPs sample. It displayed bands 3424 cm^−1^ (O-H of H_2_O molecule’s intermolecular interaction with the surface of TiO_2_), 2922, 2852 cm^−1^ (C-H stretching vibration^50^, 1626 cm^−1^ (O–H bending mode of adsorbed H_2_O molecules), 1457 cm^−1^ (C-O bond stretching from the plant extract^51^, 541 –465 cm^−1^ (O–Ti–O stretching vibration^52^. These observed bands are consistent with previously reported values for TiO_2_-NPs, further validating their structural composition^52^. A thorough analysis of these spectra offers important insights into the functional groups present and confirms the successful synthesis of TiO_2_-NPs using the *Vitis vinifera* extract. Yitagesu et al.^45^ reported a strong absorption peak in the FTIR spectrum of TiO_2_synthesized using *Impatiens rothii* Hook.f. leaf extract, appearing in the range of 850–420 cm⁻¹ and attributed to the stretching vibrations of the O–Ti–O bond. The FTIR spectrum of TiO_2_ NPs synthesized through the green method using jasmine flower extract exhibited strong bands at 460 cm⁻¹ and 900 cm⁻¹, corresponding to Ti–O and Ti–O–Ti bending vibrations, respectively^46^.

**Fig. 3:**
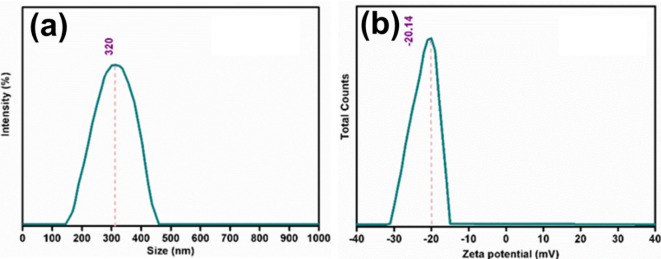
(**a**) DLS Particle size distribution, and (**b**) zeta potential of TiO_2_-NPs.

The particle size analysis and the zeta potential of TiO_2_-NPs are shown in Fig. 3. The dynamic light scattering (DLS) analysis demonstrated a well-defined particle size distribution centered at 320 ± 7.8 nm (Fig. 3a), with the intensity plot showing a symmetrical, ranging approximately from 150 to 450 nm with Polydispersity Index (0.211 ± 0.01). The zeta potential measurement exhibited a clear negative value of -20.14 ± 1.2 mV, with a single sharp peak in the distribution curve indicating uniform surface charge properties (Fig. 3b). The synthesized TiO_2_ NPs using *Tinospora cordifolia* plant extract exhibited an average hydrodynamic radius of 153.4 nm and a zeta potential of − 28.4 mV^53^. TiO_2_ NPs synthesized using *Aloe vera* leaf extract exhibited a zeta potential of − 34.1 ± 5 mV^54^. The TiO_2_ NPs synthesized via green synthesis using *Ocimum sanctum* leaf extract exhibited a zeta potential of − 11.5 mV^55^.

**Fig. 4:**
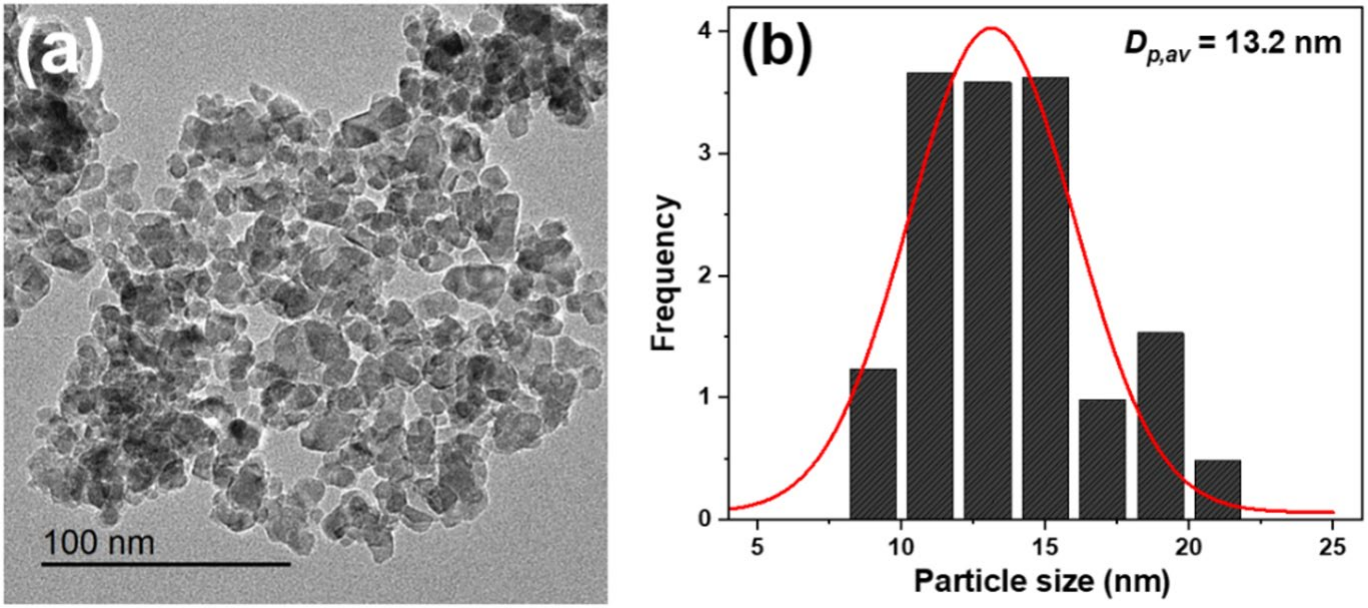
(**a**) TEM image, and (**b**) particle size distribution of TiO_2_-NPs.

The morphology and size distribution of the TiO_2_-NPs were analyzed using TEM, as illustrated in Fig. 4. The TEM image confirmed a quasi-spherical shape for the nanoparticles (Fig. 4a), while the size distribution analysis (Fig. 4b) revealed an average particle size of 13.2 nm. The consistent particle size indicates efficient synthesis and stabilization of the nanoparticles, potentially due to the role of *Vitis vinifera*as a capping or stabilizing agent. Slight agglomeration observed may result from van der Waals forces. Anbumani et al^46^. reported that TiO_2_ NPs synthesized using *Luffa acutangula* leaf extract were hexagonally in shape, with sizes ranging from approximately 10 to 49 nm. The TEM image of TiO_2_ NPs synthesized through the green method using *Echinops echinatus* plant extract revealed irregular, aggregated spherical particles with an average size of approximately 47 nm^49^.

**Fig. 5:**
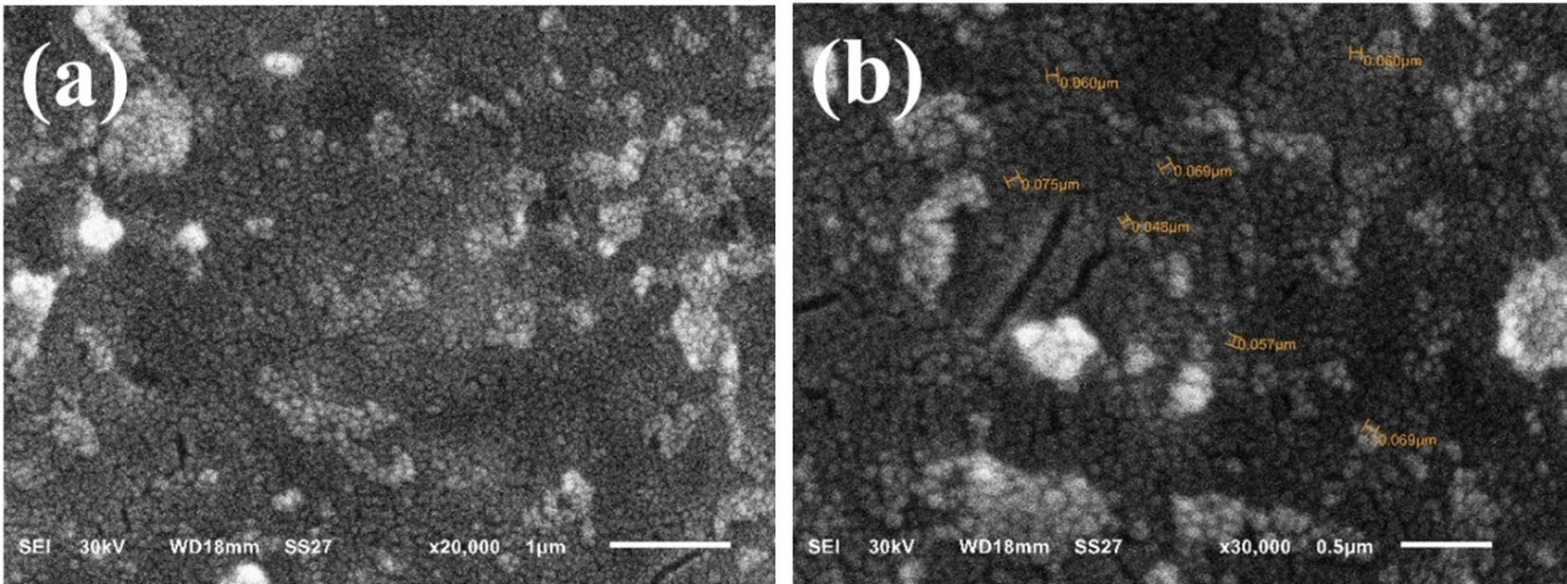
(**a**) SEM image of experimental composite resin with 10% TiO_2_ NPs. (**b**) SEM image of experimental composite resin with 20% TiO_2_ NPs.

The SEM image of experimental composite resin with 20% TiO_2_ NPs (Fig. 5) shows a granular surface morphology with evenly distributed particles (Fig. 5a). This suggests good dispersion of TiO₂ NPs in the resin matrix. At higher magnification, some regions appear denser than others, potentially indicating slight agglomeration of nanoparticles (Fig. 5b).

### Evaluation of experimental composite

#### Antimicrobial activity results

**Fig. 6:**
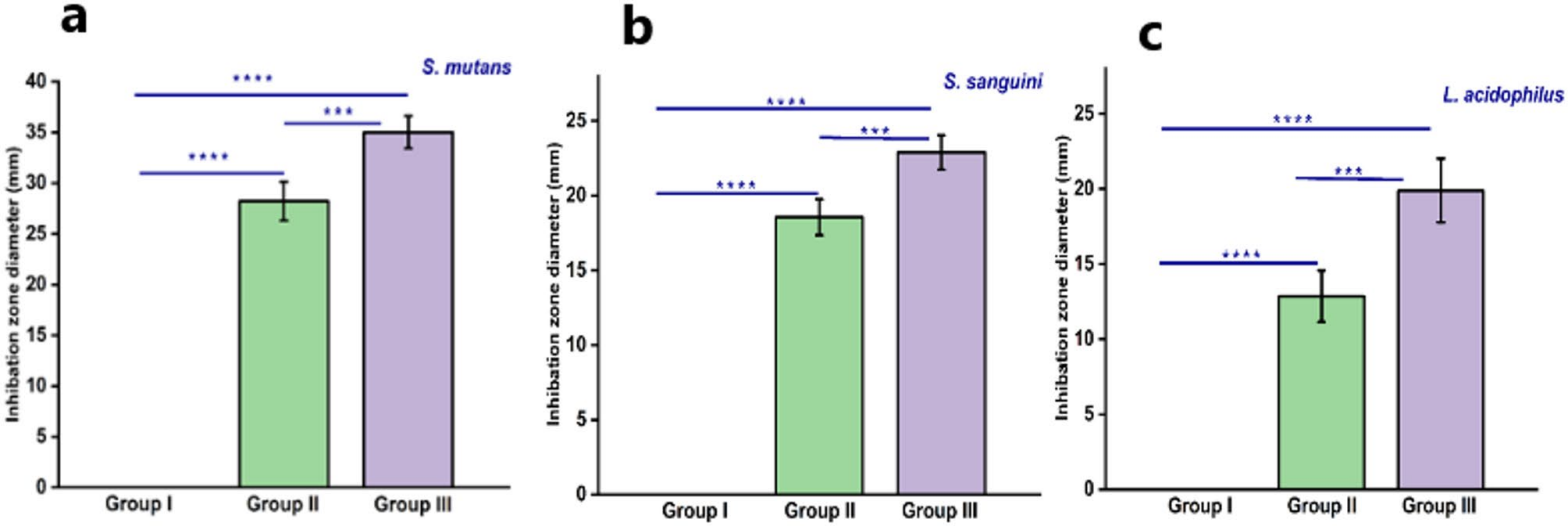
Inhibition zone diameters (mm) of unmodified composite resin (Group I), composite with 10% TiO₂ nanoparticles (Group II), and composite with 20% TiO₂ nanoparticles (Group III) against **(a)** *S. mutans*,**(b)** *S. sanguinis*, and **(c)** *L. acidophilus*. Data is presented as mean ± standard deviation, with significant differences between groups indicated (***p < 0.001, ****p < 0.0001).

The antimicrobial activity of the composite resins without and with 10% and 20% TiO_2_-NPs against *S. mutans*, S.*sanguinis*, and *L. acidophilus* was evaluated by measuring the inhibition zone diameters (mm) after 24 h. of incubation. The unmodified composite resin (Group I) exhibited the smallest inhibition zones for all three bacterial species (Fig. 6a, b and c). Incorporation of 10% TiO₂ NPs (Group II) significantly increased the inhibition zone diameters compared to Group I, indicating enhanced antibacterial activity. The composite containing 20% TiO₂ nanoparticles (Group III) demonstrated the largest inhibition zones against all tested bacteria, with *S. mutans* showing inhibition zones approaching 35 ± 1.58 mm, *S. sanguinis* around 22 ± 1.5 mm, and *L. acidophilus* about 19.88 ± 2.13 mm. Statistical analysis (One-Way Anova) revealed highly significant differences between all groups for each bacterial strain (***p < 0.001, ****p < 0.0001), confirming that increasing TiO₂ NPs concentration in the composite resin led to a dose-dependent enhancement of antibacterial efficacy.

#### Molecular docking analysis

Molecular docking analysis demonstrated that the green-synthesized titanium dioxide nanoparticles (TiO₂-NPs), modeled as a representative anatase TiO₂ cluster, exhibited a binding affinity score of − 6.12 kcal/mol against the catalytic domain of *Streptococcus mutans* glucosyltransferase (GtfC). The docking results revealed that TiO₂-NPs formed three strong ionic interactions with the catalytic residues Asp451, Asp562, and Glu489, in addition to a π-anion interaction with Leu407. Notably, Asp451 is a critical residue involved in the catalytic mechanism of GtfC, playing a key role in sucrose hydrolysis and subsequent biofilm formation.

**Fig. 7:**
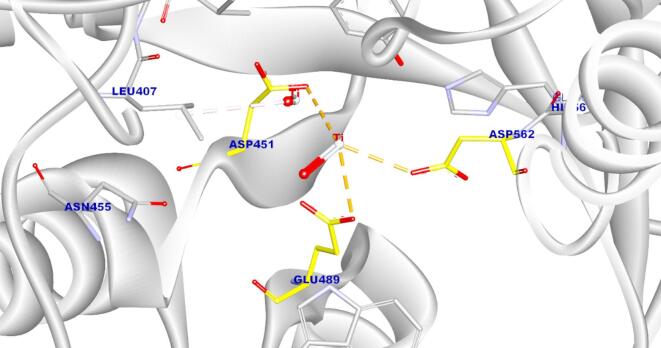
3D molecular docking visualization showing the interaction of TiO₂-NPs (gray sphere) with key residues of *S. mutans* glucosyltransferase (Asp451, Asp562, Glu489 in yellow).

These interactions suggest that TiO₂-NPs may competitively inhibit GtfC by binding to its catalytic domain, potentially displacing the natural substrate (e.g., sucrose) and disrupting glucan synthesis, which is essential for bacterial adhesion and biofilm development (Fig. 7). This molecular insight supports the observed antimicrobial activity of TiO₂-NP-modified composites against *S. mutans*.

#### Vicker micro-hardness (VHN) results

**Fig. 8:**
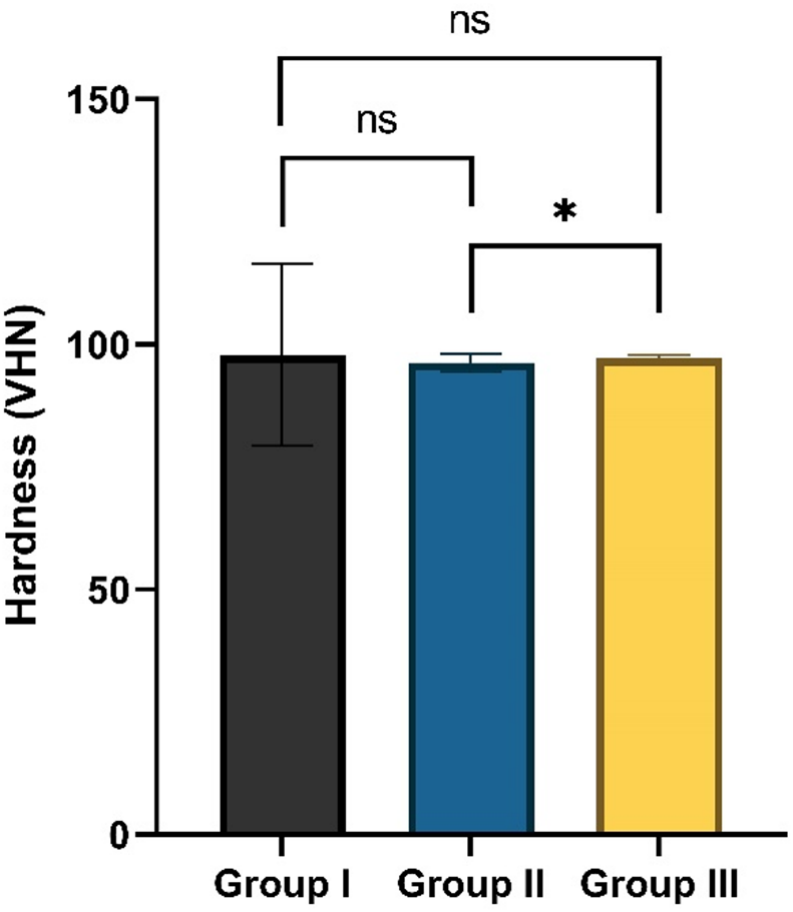
Vickers hardness (VHN) values for Group I (unmodified composite), Group II (composite containing 10% TiO_2_-NPs), and Group III (composite containing 20% TiO_2_-NPs). Asterisk (*) indicates significance (*p* < 0.05); ns: not significant (*p* > 0.05).

Microhardness values are reported in Fig. 8. As the data non-normally distributed, the Kruskal-Wallis test was used to compare the median hardness between groups and identified a statistically significant difference (*p* = 0.034). The data showed that Group III exhibited the highest median VHN with a value equal to 97.23 (0.67). Post-hoc pairwise comparisons showed a significant difference in hardness between Group II and Group III (*p* = 0.028). However, no significant differences were found between Group I and Group II (*p* = 0.475) or between Group I and Group III (*p* = 0.704).

#### Flexural strength results

**Fig. 9:**
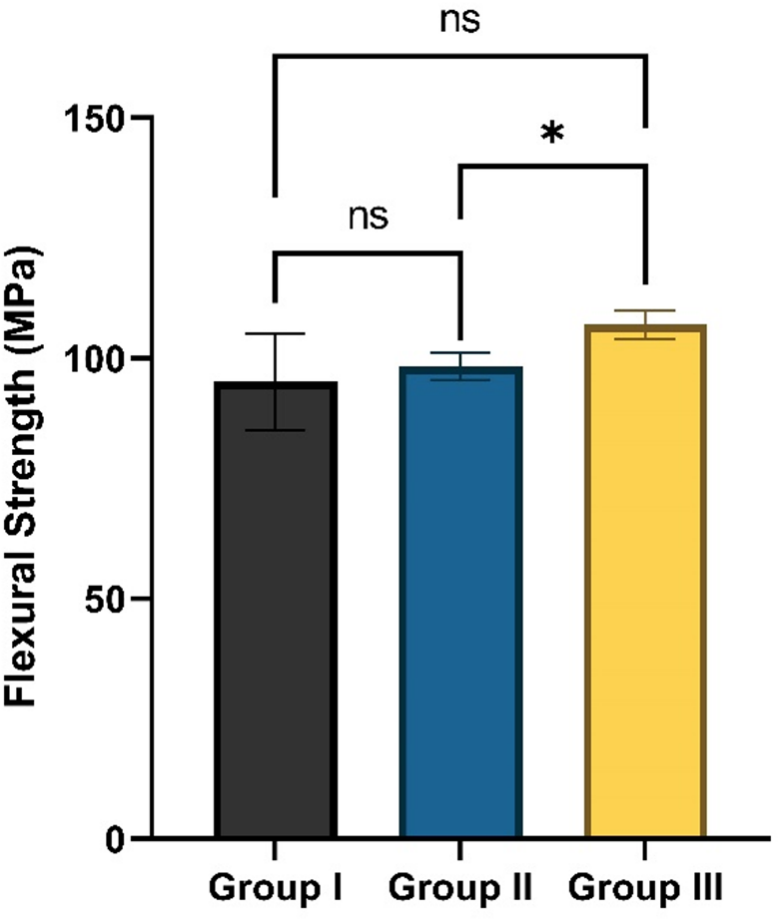
Flexural strength (MPa) values for Group I (unmodified composite), Group II (composite containing 10% TiO_2_-NPs), and Group III (composite containing 20% TiO_2_-NPs). Asterisk (*) indicates significance (*p* < 0.05); ns: not significant (*p* > 0.05).

Flexural strength values for all groups are presented as mean ± SD in Fig. 9. As the data exhibited unequal variances, Welch’s ANOVA was applied. Group III demonstrated the highest mean value (106.97 ± 2.88 MPa). Statistical analysis revealed a significant difference among the groups (*p* = 0.005) and pairwise comparisons indicated that the significant difference occurred specifically between Group II and Group III (*p* = 0.004). However, no significant differences were observed between Group I and Group II (*p* = 0.786) or between Group I and Group III (*p* = 0.117).

#### Post gel polymerization shrinkage strain results

**Fig. 10:**
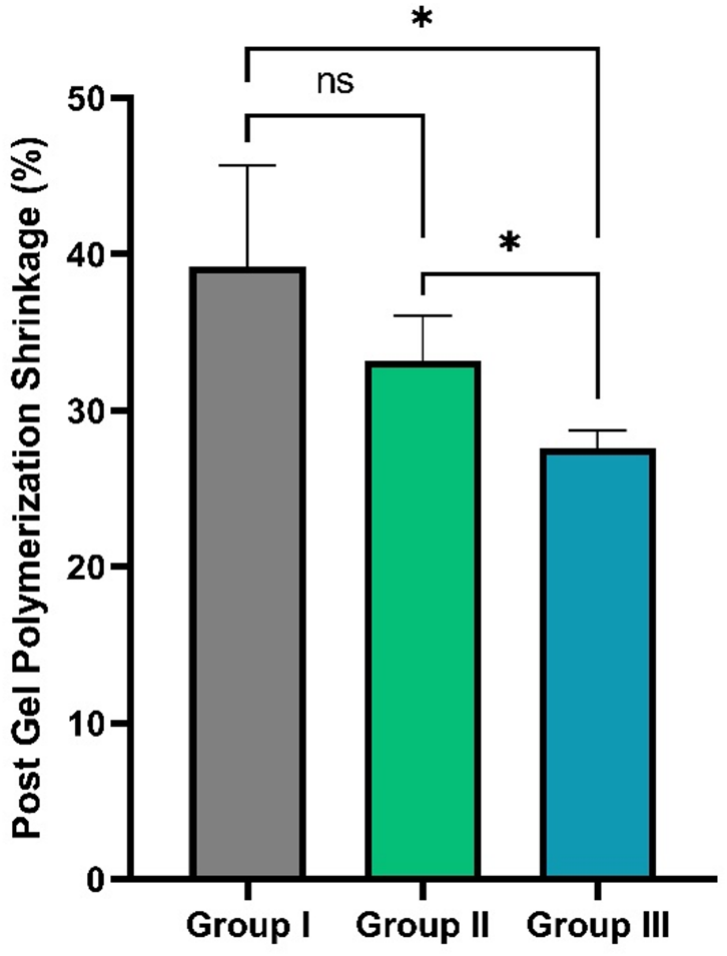
Polymerization shrinkage (%) of composite groups. Bars represent mean ± standard deviation. Group I: Control (fumed silica only); Group II: 10% green-synthesized titanium dioxide nanoparticles (TiO₂-NP); Group III: 20% TiO₂-NP. Asterisk (*) indicates significance (*p* < 0.05); ns: not significant (*p* > 0.05).

Polymerization shrinkage values for all composite groups are presented as mean ± standard deviation (SD) and visually compared in Fig. 10. As the data exhibited unequal variances, Welch’s ANOVA was applied. The control group exhibited the highest polymerization shrinkage, while both experimental groups containing green-synthesized TiO₂-NPs demonstrated significantly reduced shrinkage. Group III (20% TiO₂-NPs) showed the lowest mean polymerization shrinkage strain (27.60 ± 1.14). Statistical analysis revealed a highly significant difference among all groups (*p* < 0.005). Both modified groups had significantly lower shrinkage than the control (*p* = 0.001). Pairwise comparisons indicated significant differences between Group II and Group III (*p* = 0.020) and between Group I and Group III (*p* = 0.033), whereas no significant difference was observed between Group I and Group II (*p* = 0.228).

## Discussion

### Characterization and preparation of green-synthesized TiO₂ nanoparticles and composite resin

The present study investigates the development of a novel light-cured composite resin modified with TiO_2_-NPs synthesized from *Vitis vinifera* extract. This research highlights the potential of eco-friendly nanotechnology in enhancing dental materials, specifically in terms of antimicrobial properties, mechanical performance, and reduction of polymerization shrinkage. The use of *Vitis vinifera* extract in the green synthesis of TiO_2_-NPs offers an environmentally friendly alternative to traditional methods, eliminating the need for toxic chemicals.

The extract acts as a stabilizing agent, facilitating the synthesis of TiO_2_-NPs with a small diameter. The GC-MS analysis of *Vitis vinifera* extract identified a wide range of bioactive compounds that contribute to its potential as reducing and stabilizing agents in the green synthesis of TiO_2_-NPs. The phenolic compounds in the extract donate electrons to titanium precursors, effectively reducing them to form TiO_2_-NPs^56^. Fatty acids coat the surfaces, providing stability and preventing agglomeration, while aromatic compounds enhance this stability by forming protective layers around the nanoparticles^57^.

The successful green synthesis of TiO_2_-NPs was confirmed through various methods. The XRD result confirmed the formation of tetragonal anatase TiO_2_-NPs with an average crystallite size of 12.7 nm. The detection of bands at 541–465 cm^−1^ (O–Ti–O stretching vibration) also proved the successful synthesis of TiO_2_-NPs using the *Vitis vinifera* extract. In addition, the TEM analysis confirms the successful formation of semi-spherical nanoparticles with an average diameter of 13.2 nm, which aligns with the study by Bahari et al.^58^. The narrow size distribution further confirms the uniformity of the synthesized TiO_2_-NPs. The zeta potential of the synthesized TiO_2_-NPs was found to be equal − 20.14 mV, which suggests moderate colloidal stability, indicating sufficient electrostatic repulsion between particles to prevent aggregation, although it falls slightly below the ideal stability threshold of ± 30 mV. It was reported that the formation of anatase phase of TiO_2_-NPs plays an important role in its photocatalytic activity and antimicrobial properties^26,59^. It was found that the average particle size obtained from the DLS method was found to be larger than those estimated from TEM images. This difference arises because, unlike microscopy techniques, laser scattering does not measure the size of individual particles within aggregates. As a result, the hydrodynamic radius determined by laser scattering is often significantly larger sometimes by an order of magnitude than the actual particle sizes observed through TEM^60^.

### Antimicrobial activity

The incorporating TiO₂ nanoparticles into composite resin significantly enhances its antibacterial properties against key oral pathogens, with the effect being concentration dependent. The unmodified composite resin (Group I) showed minimal antimicrobial activity, while the addition of 10% TiO₂ nanoparticles (Group II) produced a marked increase in inhibition zones for *S. mutans*,* S. sanguinis*,* and L. acidophilus*, in agreement with previous studies reporting significant reductions in colony counts and improved biofilm inhibition when TiO₂ nanoparticles are incorporated into dental composites^61^.

The further increase to 20% TiO₂ nanoparticles (Group III) resulted in the greatest antibacterial effect, suggesting that higher nanoparticle loading can maximize microbial inhibition, likely due to increased disruption of bacterial cell walls and enhanced generation of reactive oxygen species^20^. While some earlier research indicated that *L. acidophilus* is more resistant to nanoparticle-mediated inhibition, the present results show significant antimicrobial activity even against this species at higher TiO₂ concentrations. These outcomes support the potential of TiO₂ nanoparticle-modified composites for reducing the risk of secondary caries and biofilm formation in restorative dentistry. However, it is important to balance antibacterial efficacy with other material properties, such as mechanical strength and esthetics, when considering clinical applications.

This enhancement can also be attributed to the inherent antimicrobial properties of the TiO_2_-NPs with small particle sizes which are formed and controlled by the bioactive compounds present in the *Vitis vinifera* extract. The reactive oxygen species generated by TiO_2_-NPs can disrupt bacterial membranes and biofilm formation. This enhanced efficacy can be also attributed to the particle size controlled and the small particle size of TiO_2_-NPs by the bioactive compounds in *Vitis vinifera*^62^.

Clinically, these antimicrobial enhancements could reduce the risk of secondary caries, one of the leading causes of restoration failure. By preventing bacterial colonization at restoration margins, TiO₂-NP-modified composites may extend restoration longevity and improve oral health outcomes. However, future studies should include multispecies biofilm models and in vivo evaluations to confirm these benefits under complex oral conditions.

### Molecular docking

To elucidate the potential mechanism underlying the antimicrobial activity of green-synthesized TiO₂-NPs, a representative anatase-phase cluster was constructed based on TEM analysis, following established approaches for modeling metal oxide nanoparticles in biological systems. The docking protocol utilized AutoDock Vina, with the search grid centered on the active site of GtfC as defined by co-crystallized ligands and key catalytic residues. This strategy enabled a detailed assessment of the binding interactions between the nanoparticle and the enzyme.

The observed binding affinity and specific interactions with key catalytic residues support the hypothesis that TiO₂-NPs can inhibit GtfC activity, thereby interfering with glucan synthesis and biofilm formation by *S. mutans*. This proposed mechanism is consistent with previous studies demonstrating the ability of metal oxide nanoparticles to interact with and inhibit bacterial enzymes involved in pathogenicity^63,64^. The molecular docking results thus provide a plausible explanation for the enhanced antimicrobial activity observed in the TiO₂-NP-modified composites.

These molecular docking results provide a mechanistic explanation for the antimicrobial findings reported in this study. The strong inhibition zones observed, particularly against *S.mutans*, which relies heavily on glucosyltransferase activity for biofilm development, are likely due to the binding of TiO₂-NPs to the enzyme’s active site. By blocking glucan synthesis, the nanoparticles impair the bacteria’s ability to adhere and form biofilms, ultimately enhancing the antimicrobial performance of the modified composite.

This molecular-level insight reinforces the experimental antimicrobial data and confirms that the antibacterial action of TiO₂-NPs is not merely due to general oxidative stress but also involves targeted inhibition of a key virulence factor in *S. mutans*.

However, it is important to note that this docking analysis was based on a static cluster model and did not incorporate molecular dynamics (MD) simulations or binding energy decomposition analyses. The absence of these advanced validation techniques may limit the precision of the predicted interactions and does not fully capture the dynamic nature of protein–nanoparticle interactions in biological environments. Future work should include MD simulations and experimental validation to further confirm these findings and clarify the biological relevance of the observed interactions.

### Mechanical properties

Despite the improvement in the antimicrobial activity of the composites with TiO_2_-NPs, it is very important to evaluate the mechanical properties of the composites. Flexural strength (FS) is a key indicators of mechanical performance^65^.

The addition of TiO₂-NPs also resulted in significant improvements in mechanical properties, including flexural strength and microhardness, compared to the control composites. These improvements are likely due to the uniform dispersion of nanoparticles within the resin matrix as confirmed in the SEM images, which reinforces the composite structure and enhances resistance to masticatory forces^4,66^. The FS values of all tested groups exceeded 80 MPa, aligning with ISO standards, indicating the adequate mechanical strength of the synthesized composites^67^. Also, spherical nanofillers can accommodate increased filler loads in composites^68^ .

The microhardness measurements showed no negative impact on hardness (*p* = 0.034), indicating that the structural integrity of the composite remains intact despite the addition of nanoparticles. The Vickers microhardness values for all tested groups ranged from 75 to 82 HV, which is notably higher than the ISO 4049 minimum requirement of 40 HV^67^. This enhancement in hardness is attributed to the incorporation of fumed silica in all composite groups, a component that previous studies have shown to significantly improve the hardness of nanocomposites^69^.

The findings of this study align closely with those reported by Ezzat et al.^20^, who incorporated grapefruit seed extract-mediated TiO₂ nanoparticles (GSE-TiO₂NPs) into experimental dental composites. In their work, the addition of 10 wt% and 20 wt% GSE-TiO₂NPs led to significant increases in flexural strength and modulus compared to the control group, while all groups surpassed the clinically acceptable microhardness threshold. Notably, the modified composites in both studies not only met but exceeded ISO 4049 standards for mechanical performance, underscoring the effectiveness of plant-mediated TiO₂-NP incorporation in enhancing composite durability.

The findings of this study are also consistent with those of Azmy et al.^4^, in which the effects of various nanoparticles, including chemically synthesized TiO₂-NPs, on the flexural strength of composite resins were investigated. Incorporation of TiO₂-NPs at lower concentrations (3 wt%) was found to significantly improve flexural strength due to enhanced load redistribution, whereas higher concentrations (7 wt%) resulted in a reduction in flexural strength attributed to nanoparticle agglomeration. In contrast, significant enhancements in flexural strength were observed in the present study with the incorporation of 10% and 20% TiO₂-NPs. This improvement may be attributed to the use of *Vitis vinifera* extract as a capping agent, which effectively controls nanoparticle size and prevents agglomeration, thereby allowing higher nanoparticle loadings without compromising mechanical integrity.

### Polymerization shrinkage

Polymerization shrinkage is a critical factor influencing the clinical performance of dental composite resins. Excessive shrinkage can lead to marginal gaps, microleakage, and ultimately secondary caries and restoration failure^70^. In the present study, the incorporation of green-synthesized TiO₂-NPs significantly reduced polymerization shrinkage compared to the control group. This reduction was more pronounced at higher nanoparticle loadings (20 wt% TiO₂NPs), demonstrating a dose-dependent effect.

The decrease in polymerization shrinkage can be attributed to the presence of well-dispersed TiO₂-NPs within the resin matrix, which likely restricts polymer chain mobility during curing and acts as a filler that absorbs or redistributes polymerization stresses^71^. This finding is consistent with previous studies reported that dental composites modified with plant-mediated TiO₂-NPs exhibited significantly lower polymerization shrinkage than unmodified composites, with the 20 wt% group showing the lowest shrinkage values (13.06 ± 0.92%)^20^. The similarity in outcomes across different plant-mediated TiO₂-NP systems highlights the effectiveness of green-synthesized nanoparticles in enhancing the dimensional stability of dental composites.

While this study provides comprehensive insights into the antimicrobial, mechanical, polymerization, and molecular properties of green-synthesized TiO₂-NP-modified dental composites, there remain valuable opportunities for further research. Future investigations could expand on these findings by evaluating color stability, wear resistance, and cytocompatibility under simulated oral conditions to better approximate clinical performance. Additionally, extending antimicrobial assessment to multi-species biofilm models and conducting in vivo studies will help confirm the long-term applicability and safety of these materials. From a molecular perspective, further computational analyses-such as molecular dynamics simulations and binding energy decomposition-could provide deeper understanding of the dynamic interactions between TiO₂ nanoparticles and key bacterial enzymes. Collectively, these directions will support the continued development and translational potential of eco-friendly, bioactive dental composites.

## Conclusion

The incorporation of green-synthesized TiO₂-NPs into dental composites significantly enhanced antimicrobial efficacy, mechanical properties, and polymerization shrinkage. Molecular docking analysis further revealed that TiO₂-NPs strongly interact with *S. mutans* glucosyltransferase, potentially inhibiting bacterial adhesion and biofilm formation. These findings underscore the translational potential of green TiO₂-NPs as a novel nanofiller platform for dental composites. The study provides the first evidence of their dual role in enhancing performance and interacting at the molecular level with bacterial targets, offering new insights into green nanotechnology’s role in combating dental biofilms through targeted enzyme inhibition. Future research should focus on comprehensive vivo evaluations to confirm the antimicrobial and mechanical benefits under clinical conditions, as well as long-term studies assessing bioactivity, color stability, wear resistance, and cytocompatibility in the complex oral environment will further support their safe and effective translation into dental practice.

## Supplementary Information

Below is the link to the electronic supplementary material.

Supplementary Material 1

## Data Availability

The data used to support the findings of this study are included within the article.

## Abbreviations

BIS-GMA: Bisphenol A-glycidyl dimethacrylate
TEGDMA: Triethylene glycol dimethacrylate
TIO_2_NPs: Titanium dioxide nanoparticles
MPTMS: y-Methacryloxypropyltrimethoxysilane
CQ: Camphorquinone (4,7,7-trimethylbicycloheptane-2,3-dione)
DMAEMA: 2- (N, N-Dimethylamine)-ethyl methacrylate
ASTM: American Society for Testing Materials
ISO: International Organization for Standardization
