# Supplementary material for: Antimicrobial mechanical and molecular docking analysis of dental composite resin incorporating green synthesized titanium dioxide nanoparticles from Vitis vinifera extract
Source: Sci Rep. 2025 Oct 8;15:35042. doi: 10.1038/s41598-025-20989-5 (PMC12508154; doi:10.1038/s41598-025-20989-5)
Supplement: Supplementary file 1 — Supplementary Material 1 [file 41598_2025_20989_MOESM1_ESM.docx]

The agar disc diffusion test of composite resin


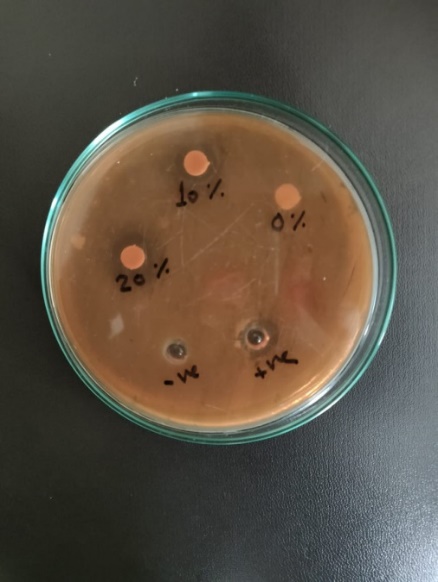


Agar dish of the antimicrobial test


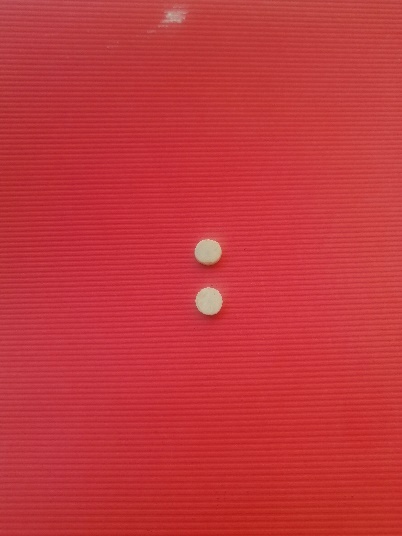

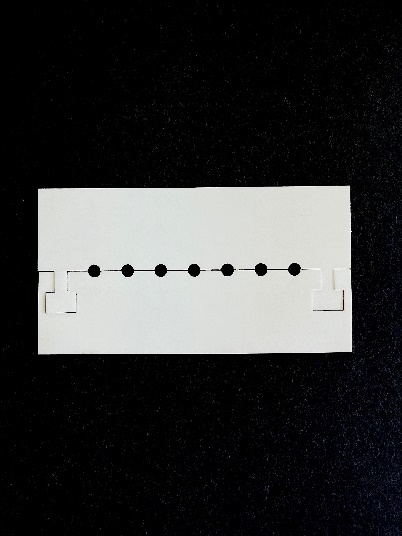

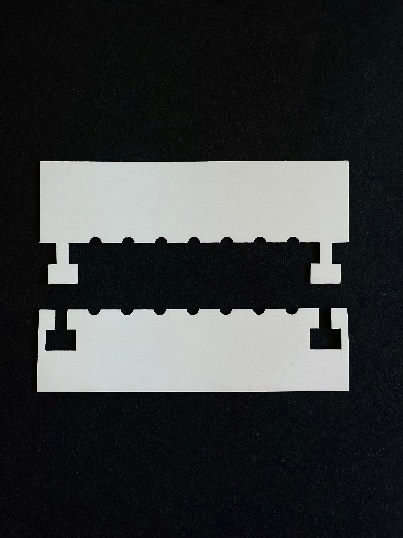


The specimens and mold for hardness test


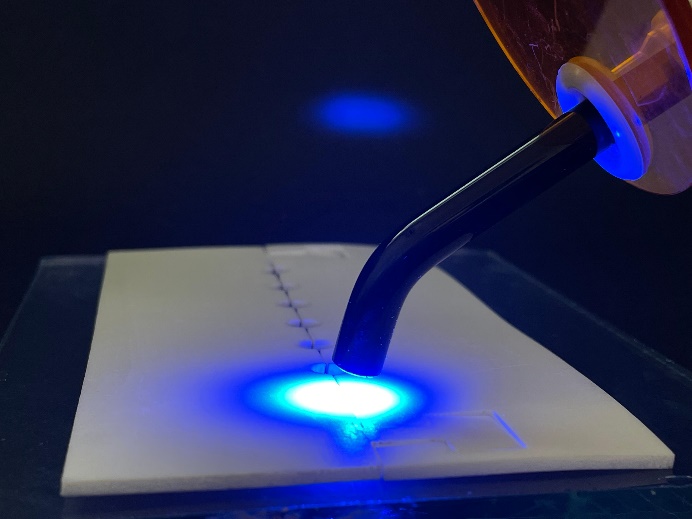


Curing for samples in polymerization shrinkage test


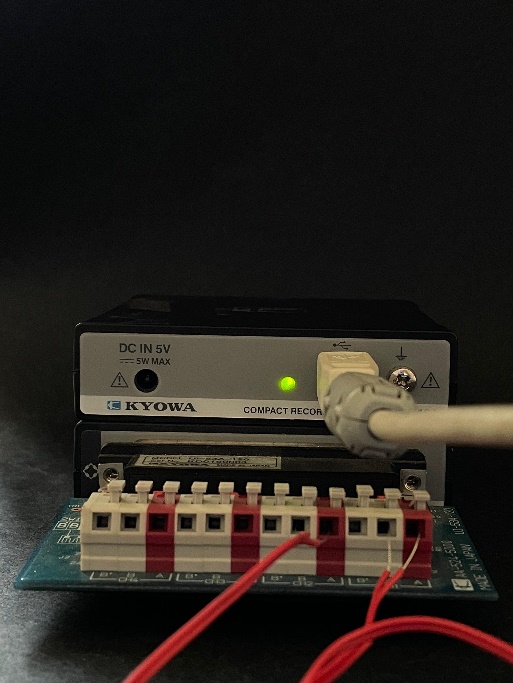

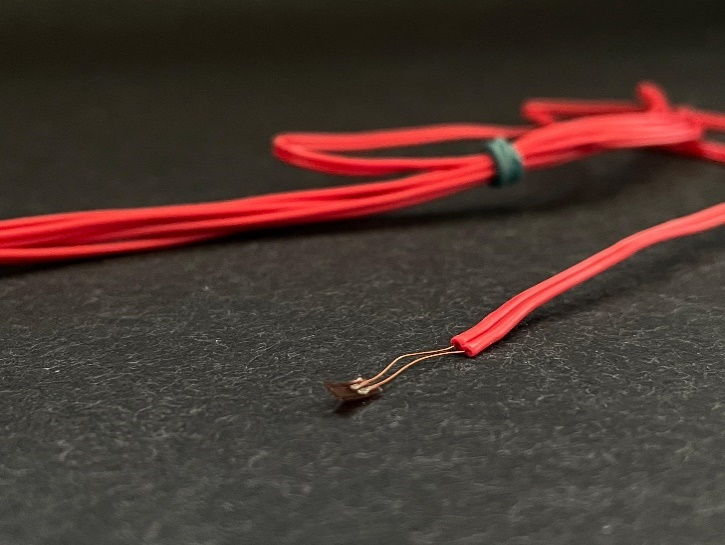


Strain gauge and strain meter


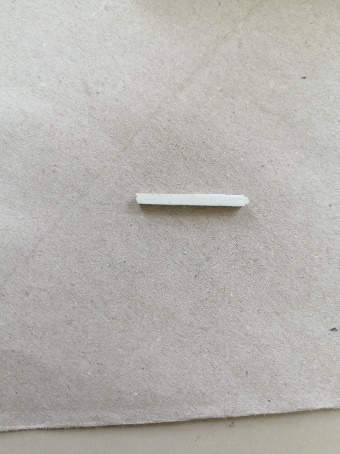

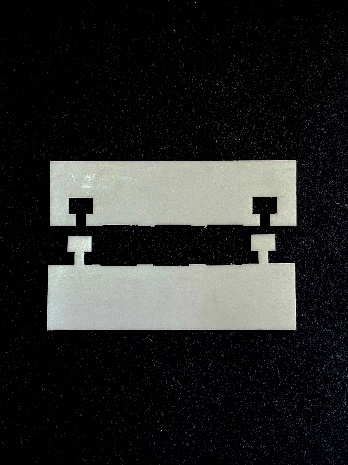

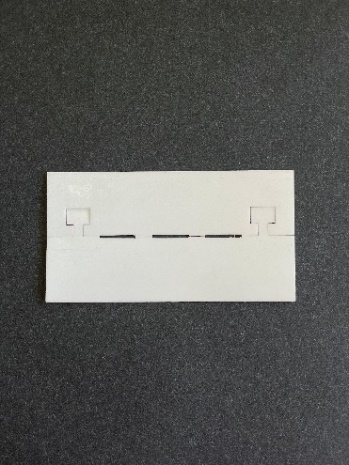


the specimen and Teflon mold of FS test


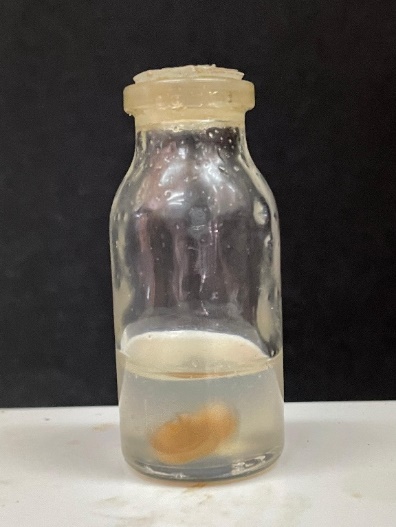

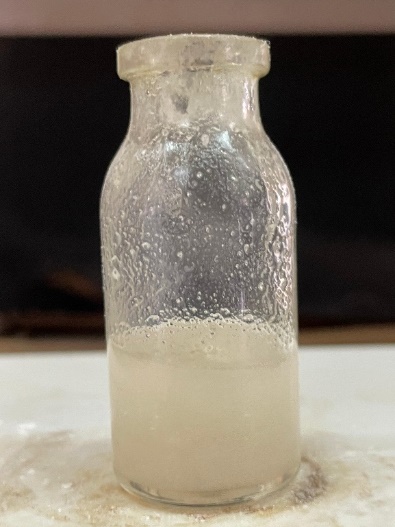


The resin matrix before and after filler incorporation
